# Predictive Value of the CT-Based Visceral Adiposity Tissue Index and Triglyceride–Glucose Index on New-Onset Atrial Fibrillation after Off-Pump Coronary Artery Bypass Graft: Analyses from a Longitudinal Study

**DOI:** 10.31083/j.rcm2411338

**Published:** 2023-11-30

**Authors:** Zhan Peng, Rui Zhao, Yunxiao Yang, Kun Hua, Xiubin Yang

**Affiliations:** ^1^Department of Cardiovascular Surgery, Beijing Anzhen Hospital, Capital Medical University, Beijing Institute of Heart, Lung and Vessel Disease, 100029 Beijing, China; ^2^Fuwai Hospital, National Center for Cardiovascular Diseases, Chinese Academy of Medical Sciences and Peking Union Medical College, 100037 Beijing, China

**Keywords:** visceral adiposity tissue index, triglyceride—glucose index, new-onset atrial fibrillation, off-pump coronary artery bypass graft

## Abstract

**Background:**

The visceral-adiposity-tissue index (VATI) and the triglyceride-glucose (TyG) index were found to be correlated with an increased risk of cardiovascular events. However, data concerning the association between the visceral adiposity/TyG indexes and the complication of new-onset postoperative atrial fibrillation (POAF), especially in patients who had just undergone off-pump coronary artery bypass grafting (OPCABG), are rare. We explored the predictive value of the computed-tomography-based VATI and the TyG index on new-onset POAF after OPCABG.

**Methods:**

This study used longitudinal data from the cohort of 542 participants who underwent OPCABG in Beijing Anzhen Hospital since June 2017. The predictive relevance of the VATI and TyG index were evaluated through Cox proportional hazards models and receiver operating characteristic (ROC) curves. The dose‒response relationship of the VATI and TyG index with new-onset POAF was analyzed by multiple-adjusted spline regression models, and sensitivity analysis was used to explore the stability of our findings.

**Results:**

The analysis found that the highest tertile of VATI [hazard ratio (HR) 2.58, 95% confidence interval (CI) 1.12–3.45; *p* = 0.01] and TyG index (HR 2.88, 95% CI 1.76–4.71; *p* = 0.01) were significantly associated with new-onset POAF compared to the lowest tertile after full adjustment for age, sex, body mass index, c-reactive protein levels, diabetes, emergency operation, New York Heart Association (NYHA) III–IV, and left atrial diameter. The area under the ROC curve (AUC) was 0.897 (*p* < 0.001) and 0.878 (*p* < 0.001) for the VATI and TyG index, respectively. In addition, the multiple-adjusted spline regression models showed a nonlinear relationship between new-onset POAF and VATI and TyG index (*p* for nonlinearity <0.001). Sensitivity analyses confirmed that the results were similar for most tertiles.

**Conclusions:**

The VATI and TyG index were significantly associated with an increased risk for the development of new-onset POAF after OPCABG.

**Clinical Trial Registration:**

NCT03729531, https://beta.clinicaltrials.gov/study/NCT03729531.

## 1. Introduction

Off-pump coronary artery bypass grafting (OPCABG) is a major treatment approach for complicated and advanced coronary artery disease [1, 2]. OPCABG avoids the negative influence of cardioplegia on the myocardium tissue, ischemia-reperfusion damage, and systemic inflammatory response from on-pump coronary artery bypass grafting (CABG) [3]. Despite its value, there is a high risk of postoperative cardiac and noncardiac complications resulting from OPCABG [3, 4, 5]. The most frequently observed cardiac dysrhythmia in these patients is new-onset postoperative atrial fibrillation (POAF) [6, 7]. POAF carries an incidence rate of 10–40% and a peak onset at two days [4, 8]. This remains a significant challenge when considering increased hospital stays and health care costs, and most notably an increased morbidity rate [9]. Despite progress in medical and surgical treatments, the overall incidence of new-onset POAF has not significantly improved [10]. Therefore, it is a clinical imperative to identify patients who are at risk for new-onset POAF early during the perioperative period as this ensures adequate precautions and optimization of clinical outcomes.

A growing number of studies indicate that excess visceral adiposity, which promotes delivery of high doses of adipokines to the heart tissues, results in atrial inflammation and myocardial lipidosis [11]. These events are associated with an increased risk of cardiovascular disease (CVD) and mortality [12, 13, 14]. Previous studies have demonstrated that insulin resistance (IR), which leads to abnormal lipid deposition and proinflammatory states, plays a key role in individuals with cardio-metabolic disorders [15]. Nevertheless, clinical evidence regarding the association between visceral adiposity/IR and the complication of POAF after OPCABG has not yet been clearly established.

The regional distribution of visceral adiposity can be accurately quantified by computed tomography (CT) imaging, which is considered to be the gold standard for measuring visceral adiposity and is termed the CT-based visceral adipose tissue index (VATI) [16]. The triglyceride-glucose (TyG) index, which has been proposed as the common test for IR assessment, can evaluate the severity of IR by measuring the triglyceride and fasting plasma-glucose levels [17, 18], and is associated with CVD [19, 20]. Accordingly, CT-based VATI and the TyG index may be valuable indicators for new-onset POAF, and the present study was designed to investigate the association of the CT-based VATI and TyG index with the incidence of new-onset atrial fibrillation after OPCABG.

## 2. Methods

### 2.1 Study Design

**Fig. 1.**
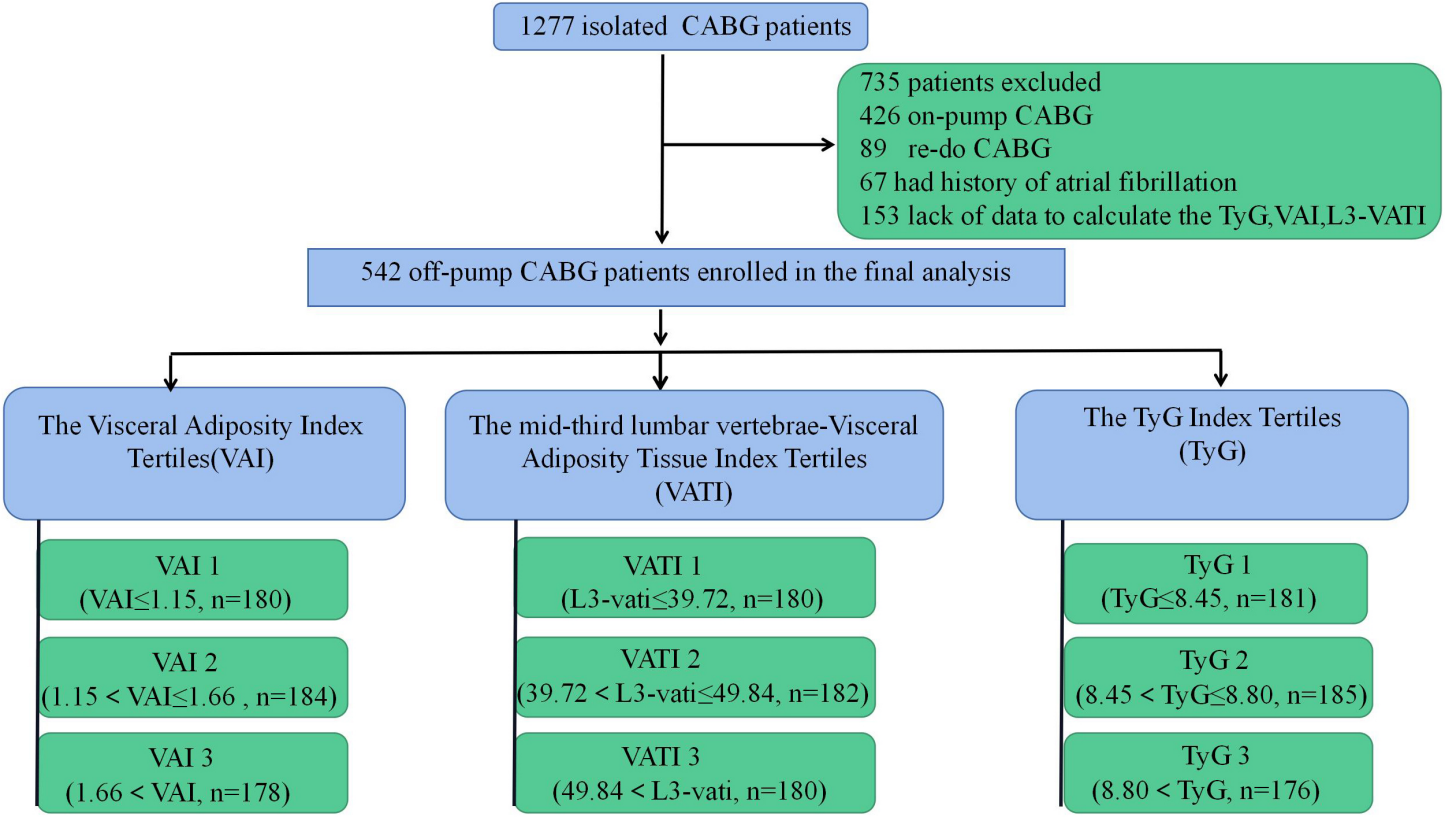
**Study flow chart**. CABG, coronary artery bypass graft; VATI, visceral adiposity tissue index; TyG, triglyceride glucose index; VAI, visceral adiposity index.

This protocol was approved by the ethics committee of Beijing Anzhen Hospital (No. 2018101X) and was drafted in accordance with the Declaration of Helsinki. Although CT-based VATI is the gold standard method to measure the level of visceral adipose tissue, the more convenient indices of visceral adiposity, the visceral adiposity index (VAI), was also calculated using waist circumference (WC), body mass index (BMI), triglycerides and high-density lipoprotein cholesterol (HDL-C) [21]. VAI measurements were also included in this study to further validate clinical evidence. From June 2017 to July 2022, all patients who received isolated CABG at the Beijing Anzhen Hospital were enrolled in this retrospective study, and the study sample was also drawn from one ongoing randomly selected longitudinal study (NCT03729531) which enrolls patients who underwent OPCABG to evaluate the effect of a no-touch saphenous vein harvesting technique with treatment details that have been previously reported previously [22]. There were 569 patients from the above longitudinal cohort, and 708 additional consecutive CABG patients, enrolled in this study resulting in a total of 1277 initial patients who underwent isolated CABG at Beijing Anzhen Hospital enrolled in this study. However, among these patients, 735 patients were excluded. Exclusion criteria included: (1) Patients receiving on-pump CABG. (2) Patients who had a history of atrial fibrillation. (3) Patients repeated CABG. (4) Patients who lacked data necessary to calculate VATI and TyG indices. Following these efforts, 542 patients that from the ongoing longitudinal cohort were deemed eligible for the present study and were divided into three groups depending on VATI, TyG index, and VAI tertiles (Fig. 1). Perioperative clinical data and blood biochemical indicators were collected from the electronic medical database system. The data of VAI and TyG were collected within 3 days prior to surgery, and CT-based VATI was obtained from CT imaging conducted during hospitalization prior to surgery.

### 2.2 Definition of the Index and Clinical Outcome

**Fig. 2.**
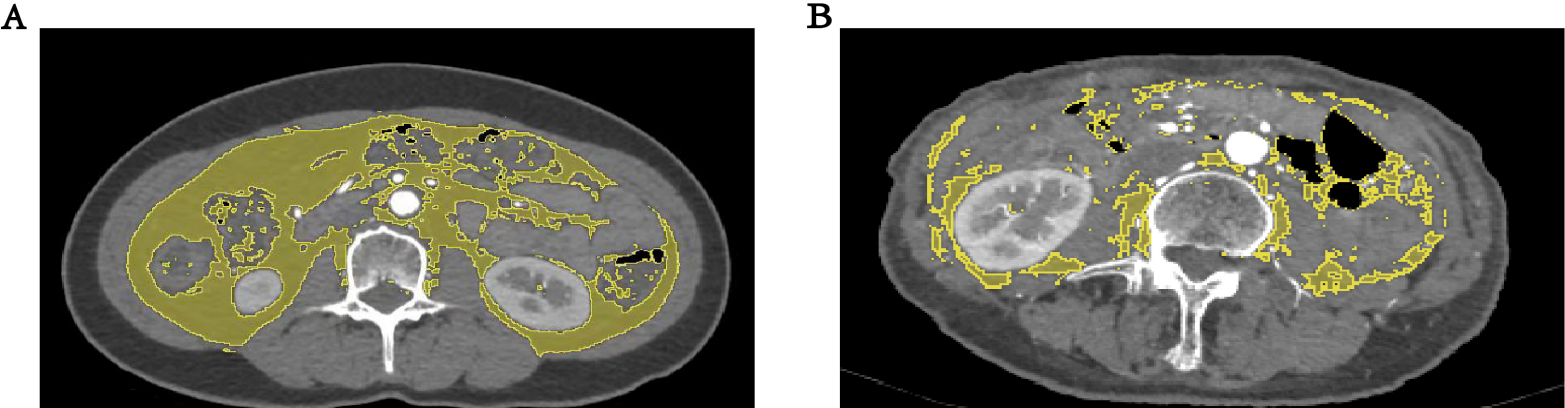
**Computed tomograph scan shows the body composition of individuals with visceral obesity**. (A) represents patients with visceral obesity, (B) represents patients without visceral obesity. Visceral adipose tissue area is highlighted in yellow.

Cross-sectional CT images in DICOM format were obtained from the picture archives and communication system within the Department of Radiology. Visceral adipose tissue area was measured using Hounsfield unit thresholds of –150 to –50 and quantified automatically using SliceOmatic (V5.0, Tomovision, Magog, Canada) software with highlighting in yellow [21] (Fig. 2). CT-based visceral adipose tissue index was defined as the cross-section of the mid-third lumbar vertebrae visceral adipose tissue area normalized for height squared (VATI, ${\mathrm{cm}}^{2}$/$m^{2}$) [21]. The calculated TyG index = ln [fasting TG (mg/dL) × fasting plasma glucose (mg/dL)/2] [17]. VAI = WC/(39.68 + 1.88 × BMI) × (triglycerides/1.03) × (1.31/HDL-C) for men, VAI = WC/(36.58 + 1.89 × BMI) × (triglycerides/0.813) × (1.52/HDL-C) for women [21].

Surgical mortality was defined as death within 30 days, or within the same hospitalization period after the surgery. Prolonged ventilator support for more than 48 h, or pneumonia, was considered a respiratory complication. The need for continuous renal replacement therapy was defined as renal dysfunction. Stroke was defined as a focal neurological symptom with rapid onset, lasting at least 24 h. Myocardial infarction (MI) associated with CABG was defined as a creatine kinase-MB measurement ≥5 times the upper limit of normal, with either new pathological Q waves, angiographic evidence of graft occlusion, native coronary artery occlusion, or evidence obtained by imaging of new loss of viable myocardium. POAF is defined as atrial fibrillation (AF) episodes lasting >30 seconds captured on a continuous wireless rhythmic monitor or electrocardiogram (ECG) monitor during the period from immediately after surgery to discharge [6].

### 2.3 Surgical Techniques and Medical Treatment

The surgical approach used at our center for off-pump CABG was to perform a median full sternotomy followed by saphenous vein, internal mammary artery, or radial artery grafting at the lesion vessels sequentially, or separately. The quality of anastomosis was routinely assessed using a transit-time flow probe (Medistim Butterfly Flowmeter, Oslo, Norway), and if the measured pulsatility index was ˂5, re-anastomosis was performed. After surgery, patients were transferred to the intensive care unit, and electrocardiographic monitoring was used to obtain daily recordings. In addition, continuous wireless rhythm monitoring, or ECG monitoring, was performed on patients who were transferred from the intensive care unit. Intravenous beta-blockers were used primarily in patients who developed new-onset POAF, and for nonresponsive patients treated with intravenous amiodarone. If a patient had been in an unstable hemodynamic status, or in POAF for more than 48 h, electrical cardioversion was performed after exclusion of the left atrial thrombus. Aspirin, clopidogrel, statins, nitrates, and bridging low-molecular-weight heparin were routinely administered during the postoperative period.

### 2.4 Statistical Analysis

Continuous variables are represented as the mean with standard deviation (SD), and non-normally distributed continuous data are expressed as the median with interquartile ranges. The Kolmogorov-Smirnov test was used for evaluation of distribution normality. A one-way analysis of variance (ANOVA) or a Kruskal-Wallis test was performed to analyze the differences in continuous variables between groups, depending on the distribution. Categorical variables were expressed as frequencies and proportions and were compared using Pearson’s chi-square test or Fisher’s exact probability test. The baseline characteristics and clinical outcomes of the study sample were described by tertiles of the VAI, the VATI, and the TyG index. The Cochran-Armitage trend χ^2^ test was used to test for trends across different index tertiles for categorical variables. Tests were conducted for linear trends based on variable containing median value for each tertile. Cox proportional-hazards models were performed to quantify associations between tertiles of the VAI, VATI, TyG index, and new-onset POAF with the lowest tertile as the reference group. We also established the following models to adjust for potential confounding factors: Model 1, unadjusted; Model 2, age and sex adjusted; and Model 3, Model 2 + BMI, C reactive protein (CRP) levels, diabetes, emergency operation (New York Heart Association) NYHA III–IV, and left atrial diameter adjusted. Calculated hazard ratio (HR) and 95% confidence interval (CI) are provided. The restricted cubic spline function, with two knots at each tertile cut-point for the three indexes, was used to analyze the dose-response relationship between VAI, VATI, and TyG index with new-onset POAF. The receiver operating characteristic (ROC) curves were calculated to assess the efficiency of VAI, VATI, and TyG index. Moreover, the area under curve (AUC) and ΔAUC were calculated to analyze the predictive value of the VAI, VATI, and TyG index, which were compared using the DeLong test. The cutoff values for VAI, VATI, and TyG index were also determined using AUC. We also performed multiple sensitivity analyses, specifically, participants with a left atrial diameter >40 mm were excluded first. Second, hypertension, total cholesterol, HDL cholesterol, and triglycerides were further adjusted to assess the stability of our study results. A two-tailed *p* ≤ 0.05 was considered statistically significant, and all the analyses were performed by R version 4.0.4 (R Foundation for Statistical Computing, Vienna, Austria) and SPSS version 26.0 software (SPSS Inc., Chicago, IL, USA).

## 3. Results

### 3.1 Baseline Characteristics

Table 1 provides the baseline characteristics of the study population according to different index tertiles. A total of 542 patients were included in the final analysis and the average age of the study population was 62.1 ± 3.4 years, and 147 (27.1%) patients were women. The cutoff values of the tertiles of the TyG index were 8.45 and 8.80, the cutoff values of the tertiles of VATI were 39.72 and 49.84, and the cutoff values of the tertiles of VAI were 1.15 and 1.66.

**Table 1.** **Baseline characteristics and perioperative variables according to different tertiles**.

Characteristic		Visceral Adiposity Index Tertiles				VATI Tertiles				TyG Index Tertiles			
		VAI 1 (≤1.15) (n = 180)	VAI 2 (1.15–1.66) (n = 184)	VAI 3 (>1.66) (n = 178)	*p*	VATI 1 (≤39.72) (n = 180)	VATI 2 (39.72–49.84) (n = 182)	VATI 3 (>49.84) (n = 180)	*p*	TyG 1 (≤8.45) (n = 181)	TyG 2 (8.45–8.80) (n = 185)	TyG 3 (>8.80) (n = 176)	*p*
Baseline clinical profile													
	Age (years)	63.2 ± 3.8	62.1 ± 3.7	61.1 ± 3.6	0.06	62.1 ± 3.7	62.4 ± 3.7	61.9 ± 3.8	0.84	62.4 ± 3.1	62.1 ± 2.9	61.9 ± 3.3	0.84
	Male [% (n)]	77.5 (141)	70.9 (129)	70.2 (125)	0.23	79.4 (143)	73.6 (134)	65.6 (118)	0.01	76.2 (138)	70.8 (131)	71.6 (126)	0.45
	Height (cm)	168.3 ± 6.5	167.1 ± 7.8	166.4 ± 7.7	0.05	170.3 ± 6.3	167.4 ± 6.7	164.2 ± 7.7	0.01	168.4 ± 6.9	166.7 ± 7.5	166.8 ± 7.6	0.04
	BMI (kg/$m^{2}$)	25.7 ± 3.2	26.2 ± 2.7	26.7 ± 3.5	0.01	23.8 ± 2.1	26.3 ± 2.6	28.4 ± 3.1	0.01	25.4 ± 3.1	26.1 ± 3.2	27.1 ± 3.1	0.01
	Hypertension [% (n)]	56.6 (103)	57.1 (104)	57.3 (102)	0.99	54.4 (98)	50.5 (92)	66.1 (119)	0.01	54.1 (98)	60.5 (112)	56.3 (99)	0.45
	Diabetes [% (n)]	35.6 (42)	46.0 (64)	39.4 (61)	0.21	35.2 (37)	41.2 (56)	43.3 (74)	0.41	36.3 (45)	48.1 (63)	37.6 (59)	0.10
	History of smoking [% (n)]	40.0 (72)	45.8 (82)	48.0 (83)	0.34	46.6 (82)	38.7 (70)	48.6 (87)	0.22	45.6 (82)	41.7 (75)	46.6 (82)	0.91
	COPD [% (n)]	0 (0)	0 (0)	1.1 (2)	0.79	0.6 (1)	0 (0)	0.6 (1)	0.60	0.6 (1)	0 (0)	0.6 (1)	0.59
	History of PCI [% (n)]	5.5 (10)	4.9 (9)	6.7 (12)	0.75	4.4 (8)	8.2 (15)	4.4 (8)	0.19	6.6 (12)	4.9 (9)	5.7 (10)	0.76
	History of stroke [% (n)]	11.0 (20)	12.1 (22)	14.0 (25)	0.67	15.0 (27)	12.6 (23)	9.4 (17)	0.27	11.6 (21)	15.7 (29)	9.7 (17)	0.21
	History of hyperlipemia [% (n)]	29.1 (53)	26.4 (48)	28.7 (51)	0.82	28.9 (52)	29.6 (54)	25.5 (46)	0.38	24.4 (43)	29.2 (54)	30.4 (55)	0.42
	History of Chronic heart failure [% (n)]	8.3 (15)	6.7 (12)	9.0 (16)	0.69	5.1 (9)	7.2 (13)	11.7 (21)	0.06	5.6 (10)	9.3 (17)	9.1 (16)	0.34
	NYHA III–IV [% (n)]	33.5 (61)	33.5 (61)	29.2 (52)	0.56	40.0 (72)	26.9 (49)	29.5 (53)	0.17	33.7 (61)	34.1 (63)	28.4 (50)	0.45
	Emergency operation [% (n)]	2.2 (4)	2.7 (5)	1.7 (3)	0.79	2.2 (4)	2.7 (5)	1.7 (3)	0.78	3.3 (6)	2.2 (4)	1.1 (2)	0.37
	Baseline SBP (mmHg)	136.8 ± 22.0	137.3 ± 21.4	137.1 ± 22.4	0.91	136.1 ± 22.3	137.5 ± 21.6	138.2 ± 23.7	0.82	136.5 ± 21.3	136.8 ± 21.7	137.5 ± 23.5	0.86
	Baseline DBP (mmHg)	71.9 ± 11.8	71.1 ± 11.9	71.5 ± 12.6	0.73	71.1 ± 11.9	71.1 ± 11.9	71.1 ± 11.9	0.81	71.1 ± 11.9	71.1 ± 11.9	71.1 ± 11.9	0.71
	Creatinine (umol/L)	73.5 ± 19.7	73.9 ± 20.4	75.8 ± 39.3	0.69	75.0 ± 31.4	77.1 ± 32.2	71.1 ± 17.3	0.12	74.4 ± 32.3	73.4 ± 17.9	75.1 ± 31.4	0.82
	TNI (ng/mL)	3.8 ± 11.2	2.1 ± 4.1	4.8 ± 15.7	0.15	4.3 ± 12.3	3.0 ± 10.8	3.5 ± 11.7	0.68	3.8 ± 11.2	3.6 ± 12.9	3.4 ± 10.0	0.94
	CRP (mg/L)	86.6 ± 82.2	87.9 ± 86.3	88.7 ± 86.3	0.85	85.6 ± 81.2	88.6 ± 84.2	89.1 ± 83.6	0.81	87.2 ± 81.8	87.5 ± 83.7	88.3 ± 84.8	0.79
	BNP (mmol/L)	83.0 ± 137.3	86.7 ± 125.8	94.3 ± 177.0	0.76	90.4 ± 147.1	87.2 ± 157.2	86.3 ± 139.4	0.96	80.6 ± 147.7	91.5 ± 140.0	91.8 ± 156.5	0.71
	Fasting glucose (mmol/L)	5.3 ± 1.7	5.3 ± 1.8	5.9 ± 2.6	0.01	4.9 ± 1.5	5.2 ± 1.8	6.3 ± 2.4	0.01	4.5 ± 1.1	5.2 ± 1.5	6.8 ± 2.6	0.01
	Triglycerides (mmol/L)	0.9 ± 0.3	1.4 ± 0.4	1.9 ± 0.7	0.01	1.3 ± 0.6	1.4 ± 0.5	1.6 ± 0.7	0.01	1.0 ± 0.3	1.4 ± 0.4	2.0 ± 0.7	0.01
	HDL-C (mmol/L)	1.3 ± 0.3	1.1 ± 0.2	1.0 ± 0.2	0.01	1.2 ± 0.3	1.2 ± 0.3	1.1 ± 0.2	0.01	1.2 ± 0.3	1.2 ± 0.4	1.2 ± 0.3	0.77
	LDL-C (mmol/L)	2.1 ± 0.7	2.2 ± 0.8	2.3 ± 0.7	0.13	2.2 ± 0.7	2.2 ± 0.8	2.2 ± 0.7	0.97	2.1 ± 0.6	2.2 ± 0.8	2.3 ± 0.7	0.10
	WC (cm)	78.0 ± 5.1	79.1 ± 6.3	83.7 ± 9.9	0.01	75.1 ± 3.4	78.9 ± 4.6	86.7 ± 8.8	0.01	77.8 ± 4.8	78.6 ± 6.4	84.4 ± 9.6	0.01
Angiographic and echocardiographic data													
	LVEF (%)	60 ± 7	59 ± 8	59 ± 8	0.31	59 ± 8	59 ± 8	59 ± 8	0.96	60 ± 8	59 ± 9	59 ± 7	0.68
	LVEDD (mm)	45.9 ± 5.7	45.5 ± 5.4	46.0 ± 5.2	0.68	45.8 ± 5.5	46.0 ± 5.1	45.7 ± 5.6	0.85	45.8 ± 5.6	45.6 ± 5.4	46.1 ± 5.1	0.67
	Left atrial diameter (mm)	39.4 ± 5.1	39.6 ± 5.0	38.5 ± 5.1	0.07	39.1 ± 5.1	39.7 ± 5.0	38.8 ± 5.3	0.25	39.3 ± 5.2	39.5 ± 5.4	38.7 ± 5.1	0.30
	Aneurysm [% (n)]	2.7 (5)	3.3 (6)	1.7 (3)	0.61	2.8 (5)	3.3 (6)	1.7 (3)	0.61	2.2 (4)	1.6 (3)	4.0 (7)	0.34
	LAD lesion [% (n)]	93.4 (170)	91.8 (167)	89.3 (159)	0.37	92.2 (166)	94.0 (171)	88.3 (159)	0.14	92.3 (167)	94.1 (174)	88.1 (155)	0.11
	LCX lesion [% (n)]	86.8 (158)	92.9 (169)	86.5 (154)	0.09	90.0 (162)	87.4 (159)	88.9 (160)	0.72	88.4 (160)	89.2 (165)	88.6 (156)	0.97
	RCA lesion [% (n)]	86.8 (158)	85.2 (155)	87.1 (155)	0.85	83.3 (150)	89.6 (163)	86.1 (155)	0.22	85.6 (155)	86.5 (160)	86.9 (153)	0.93
	LM lesion [% (n)]	70.9 (129)	73.6 (134)	71.3 (127)	0.82	71.1 (128)	64.8 (118)	79.9 (144)	0.06	69.6 (126)	70.8 (131)	75.6 (133)	0.41
	Multivessel lesion [% (n)]	91.2 (166)	92.9 (169)	93.3 (166)	0.73	92.2 (166)	92.3 (168)	92.8 (167)	0.97	90.6 (164)	88.6 (169)	81.3 (168)	0.17
Perioperative variables													
	Duration of operation (h)	4.1 ± 0.8	4.1 ± 0.8	4.1 ± 0.7	0.68	4.0 ± 0.7	4.1 ± 0.8	4.2 ± 0.8	0.16	4.1 ± 0.7	4.1 ± 0.8	4.2 ± 0.7	0.36
	ICU staying (h)	43.6 ± 32.4	42.0 ± 26.4	45.7 ± 33.2	0.51	42.5 ± 29.7	43.5 ± 30.3	45.2 ± 32.4	0.69	45.4 ± 31.4	41.5 ± 28.8	44.4 ± 32.1	0.45
	Time of mechanical ventilation (h)	29.7 ± 24.4	26.3 ± 15.5	29.3 ± 21.4	0.22	26.1 ± 17.5	28.5 ± 20.2	30.6 ± 24.1	0.12	30.2 ± 23.0	27.1 ± 18.9	28.1 ± 20.8	0.35
	Number of grafts	3 [1, 4]	3 [2, 5]	3 [1, 4]	0.69	3 [1, 5]	3 [1, 6]	3 [1, 5]	0.19	3 [1, 4]	3 [2, 5]	3 [1, 4]	0.34

VATI, visceral adiposity tissue index; TyG, triglyceride glucose index; VAI, visceral adiposity index; BMI, body mass index; COPD, chronic obstructive pulmonary disease; PCI, percutaneous coronary intervention; SBP, systolic blood pressure; DBP, diastolic blood pressure; CRP, C-reaction protein; TNI, troponin I; BNP, brain natriuretic peptide; HDL-C, high-density lipoprotein cholesterol; LDL-C, low-density lipoprotein cholesterol; WC, waist circumference; NYHA, New York Heart Association; LVEF, left ventricular ejection fraction; LVEDD, left ventricular end-diastolic diameter; LAD, left anterior descending artery; LCX, left circumfex artery; RCA, right coronary artery; LM, left main coronary artery; ICU, intensive care unit.

The study population was classified into nine subgroups depending on the different index tertiles for further analysis: the VAI 1 group (n = 180), the VAI 2 group (n = 184), and the VAI 3 group (n = 178); the VATI 1 group (n = 180), the VATI 2 group (n = 182) and the VATI 3 group (n = 180); and the TyG 1 group (n = 181), the TyG 2 group (n = 185) and the TyG 3 group (n = 176). Compared with the lowest tertiles of VAI, participants with higher levels of VAI were more inclined to have higher BMI, fasting glucose, triglycerides, low-density lipoprotein cholesterol (LDL-C), and WC values, and to have a lower levels of HDL-C. In addition, patients in the higher VATI tertile when compared to the lowest tertile had higher BMI, fasting glucose, triglycerides, LDL-C, and WC levels but a lower level of HDL-C. These patients were less likely to be males and had a higher proportion of hypertension. For TyG index, higher BMI, fasting glucose, triglycerides and WC levels were more likely found in the TyG 3 group than in the other tertile groups.

### 3.2 Predictive Value of the VAI, VATI and TyG Index

The relationships between VAI, VATI, TyG index and new-onset POAF are presented in Table 2 (**Supplementary Fig. 1** and **Supplementary Table 1**). In the unadjusted Cox proportional hazards models, when compared with the lowest tertile, the HRs (95% CIs) of the highest tertile of the VAI, VATI and TyG index for POAF were 2.28 (1.47–3.23), 4.23 (2.74–6.042) and 3.64 (2.36–5.62), respectively. In the age and sex-adjusted Cox proportional hazards models, when compared with the lowest tertile, the HRs (95% CIs) of the highest tertile of the VAI, VATI and TyG index for POAF were 2.08 (1.35–3.22), 3.16 (1.24–4.20) and 3.12 (1.93–5.05), respectively. After further adjustment for covariables of BMI, CRP, diabetes, emergency operation, NYHA III–IV, and left atrial diameter in Model 3, the higher VAI, VATI and TyG index were significantly associated with an increased risk of POAF. Fully adjusted HRs (95% CIs) of the VAI, VATI and TyG index in tertile 3 versus tertile 1 were 1.88 (1.21–2.95), 2.58 (1.12–3.45) and 2.88 (1.76–4.71), respectively, for POAF. In addition, in the fully adjusted model, each 1.0 SD increase in the VAI, VATI and TyG index was related to an increased risk of POAF, and the HR values were 1.14 (1.02–1.27), 1.53 (1.06–2.28) and 1.24 (1.03–1.73), respectively.

**Table 2.** **HRs and 95% CIs for POAF by different indexes tertiles**.

Model		Visceral Adiposity Index Tertiles (HRs and 95% CIs)					VATI Tertiles (HRs and 95% CIs)					TyG Index Tertiles (HRs and 95% CIs)				
		VAI 1	VAI 2	VAI 3	*p* Trend	Each 1-SD increase	VATI 1	VATI 2	VATI 3	*p* Trend	Each 1-SD increase	TyG 1	TyG 2	TyG 3	*p* Trend	Each 1-SD increase
POAF																
	Model 1	1.0	2.16 (1.19–2.29)	2.28 (1.47–3.23)	0.01	1.15 (1.05–1.27)	1.0	2.35 (1.15–2.81)	4.23 (2.74–6.042)	0.01	1.54 (1.10–2.15)	1.0	2.67 (1.82–2.39)	3.64 (2.36–5.62)	0.01	1.23 (1.09–1.70)
	Model 2	1.0	1.53 (0.87–1.86)	2.08 (1.35–3.22)	0.02	1.15 (1.04–1.23)	1.0	1.36 (1.02–1.81)	3.16 (1.24–4.20)	0.01	1.56 (1.12–2.19)	1.0	1.67 (0.92–1.89)	3.12 (1.93–5.05)	0.02	1.24 (1.04–1.71)
	Model 3	1.0	1.12 (0.49–1.73)	1.88 (1.21–2.95)	0.03	1.14 (1.02–1.27)	1.0	1.33 (0.94–1.78)	2.58 (1.12–3.45)	0.01	1.53 (1.06–2.28)	1.0	1.54 (0.25–1.17)	2.88 (1.76–4.71)	0.04	1.24 (1.03–1.73)

VATI, The mid-third lumbar vertebrae visceral adiposity tissue index; TyG, triglyceride glucose index; VAI, visceral adiposity index; CI, confidence interval; POAF, postoperative atrial fibrillation; BMI, body mass index; CRP, C-reaction protein; NYHA, New York Heart Association; HR, hazard ratio. Model 1, unadjusted; Model 2, age, sex; Model 3, Model 2 + BMI, CRP, Diabetes, Emergency operation, NYHA III–IV, and Left atrial diameter. Tests for linear trends based on variable containing median value for each tertile.

**Fig. 3.**
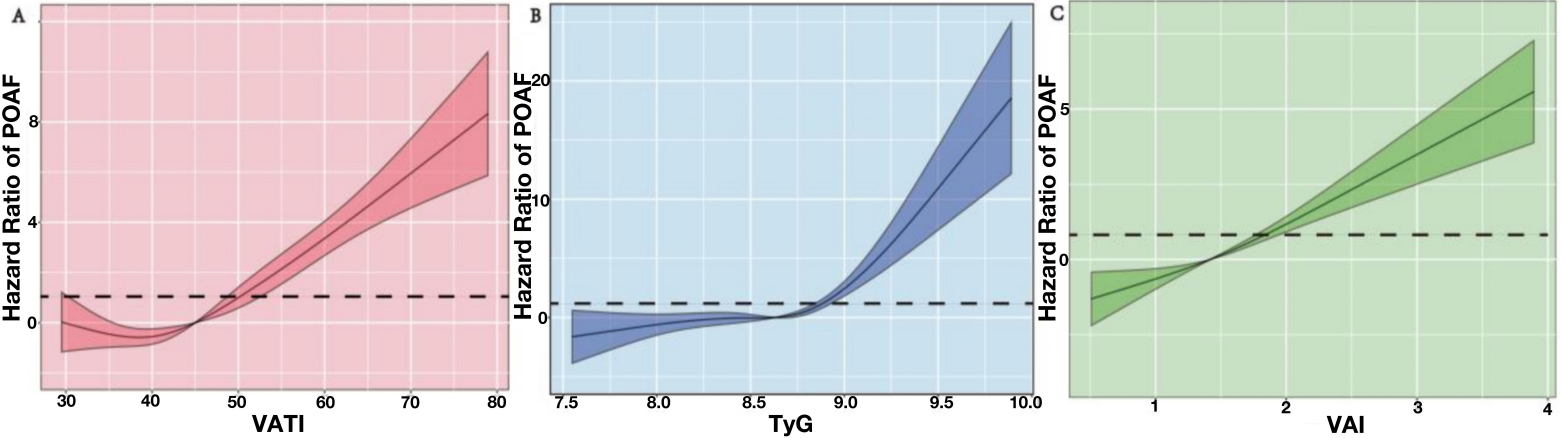
**Dose–response relationship of the VATI, VAI and TyG index with new-onset atrial fibrillation following off-pump coronary artery bypass graft**. Hazard ratios and 95% confidence intervals derived from restricted cubic spline regression, with with two knots at each tertile cut-point of the distribution of the VATI, VAI and TyG index levels. (A) The nonlinear relationship between POAF and VATI (*p* for nonlinearity <0.001). (B) The nonlinear relationship between POAF and TyG index (*p* for nonlinearity <0.001). (C) The linear relationship between POAF and VAI (*p* for nonlinearity = 0.265). VATI, visceral adiposity tissue index; TyG, triglyceride glucose index; VAI, visceral adiposity index; POAF, postoperative atrial fibrillation; HR, hazard ratios; CI, confidence intervals.

**Fig. 4.**
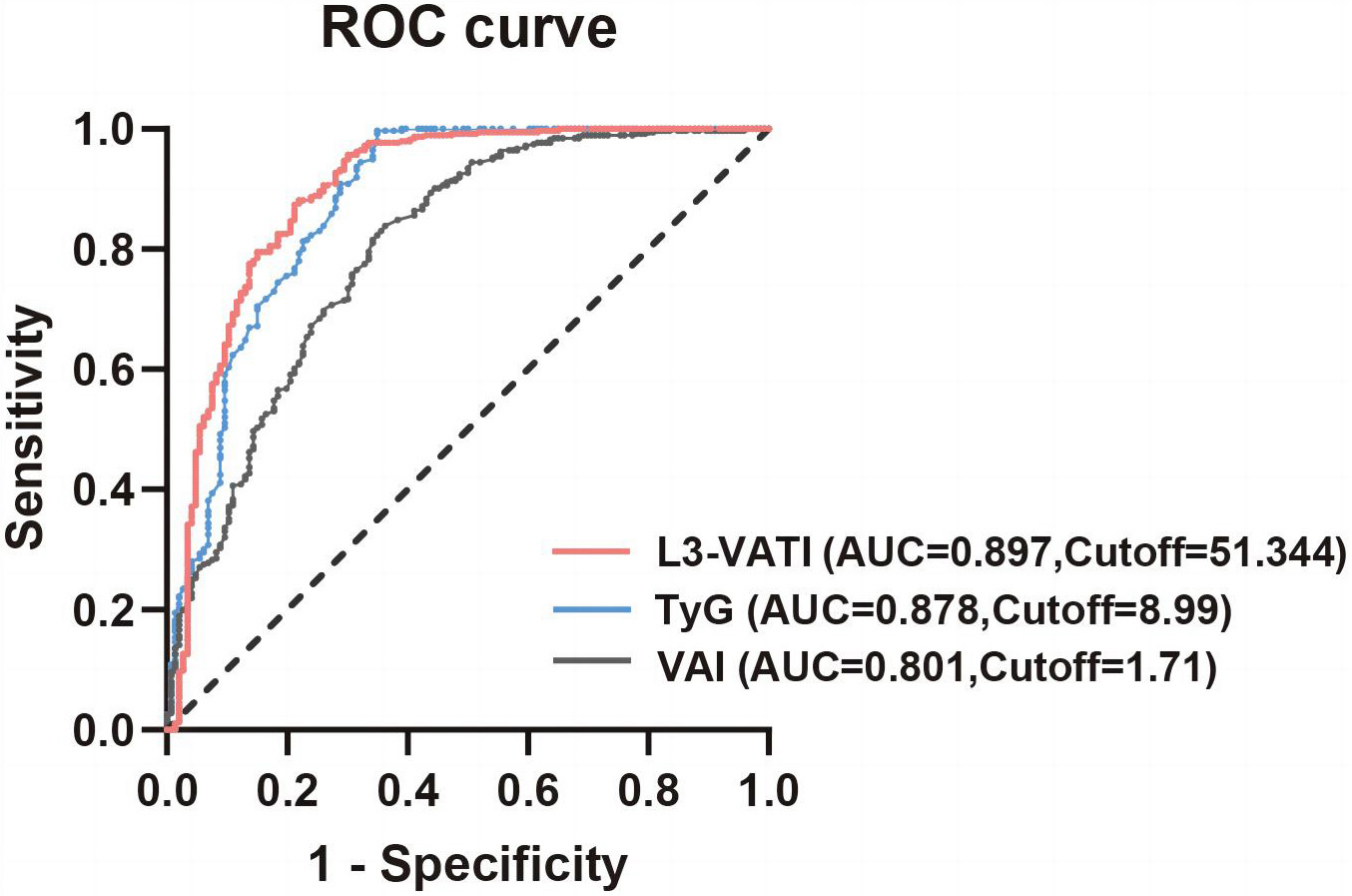
**The receiver operating characteristic (ROC) curves of the VATI, VAI and TyG index added into original model for predicting new-onset atrial fibrillation following off-pump coronary artery bypass graft**. The red solid line, ROC curves of the L3-VATI added into original model (AUC = 0.897, 95% CI: 0.861–0.916, Cutoff value = 51.34, *p* < 0.001); The blue solid line, ROC curves of the TyG index added into original model (AUC = 0.878, 95% CI: 0.840–0.896, Cutoff value = 8.99, *p* = 0.005); The black solid line, ROC curves of the VAI added into original model (AUC = 0.801, 95% CI: 0.756–0.845, Cutoff value = 1.71, *p* = 0.021). L3-VATI, The mid-third lumbar vertebrae visceral adiposity tissue index; TyG, triglyceride glucose index; VAI, visceral adiposity index; AUC, area under the curve; BMI, body mass index; CRP, C-reaction protein; NYHA, New York Heart Association; original model included age, sex, BMI, CRP, diabetes, emergency operation, NYHA III–IV, and left atrial diameter.

The dose-response relationships between TyG index, VATI, and VAI with the risk of new-onset POAF is shown in Fig. 3. Multiple-adjusted spline regression models indicate a significant nonlinear relationship between POAF and VATI (*p* for nonlinearity <0.001; Fig. 3A), and between POAF and TyG index (*p* for nonlinearity <0.001; Fig. 3B); however, there was no statistically significant nonlinearity relationship between POAF and VAI (*p* for nonlinearity = 0.265; Fig. 3C). The ROC curves of VAI, the VATI, and TyG index are presented in Fig. 4. We observed that the AUC of the original model in the total study sample for new-onset POAF was 0.794 (0.747–0.836) (Table 3). When the VAI, VATI, and TyG index were added into the original model which included age, sex, BMI, CRP, diabetes, emergency surgery, NYHA III–IV, and left atrial diameter, the DeLong test indicated that the AUC significantly improved (*p* < 0.05) for all three indexes (ΔAUC = 0.007, 0.084, and 0.103, respectively). Moreover, the optimal values of VAI, VATI, and TyG index for predicting POAF after OPCABG were 1.71, 51.34, and 8.99, respectively (Fig. 4).

**Table 3.** **Predictive value of the VAI, VATI and TyG index for new-onset postoperative atrial fibrillation**.

Model	AUC (95% CI)	∆AUC	*p*-value
Original model*	0.794 (0.747–0.836)	-	-
Original model + VATI	0.897 (0.861–0.916)	0.103	<0.001
Original model + TyG index	0.878 (0.840–0.896)	0.084	0.005
Original model + VAI	0.801 (0.756–0.845)	0.007	0.021

VATI, visceral adiposity tissue index; TyG, triglyceride glucose index; VAI, visceral adiposity index; AUC, area under the curve; BMI, body mass index; CRP, C-reaction protein; NYHA, New York Heart Association; CI, confidence interval. *Original model included age, sex, BMI, CRP, diabetes, emergency operation, NYHA III–IV, and left atrial diameter.

Sensitivity analyses were performed for VATI, VAI, and TyG index, and these confirmed that the results were similar for most tertiles (**Supplementary Table 2**). We repeated the analysis after excluding patients with a left atrial diameter >40 mm. TyG group 3 and VATI group 3 patients were significantly associated with POAF (HR: 1.52, 95% CI 1.12–2.75 and HR: 2.18, 95% CI 1.31–3.27), however, VAI group 3 was not (HR: 1.26, 95% CI 0.95–1.47). The results remained fairly consistent when hypertension, total cholesterol, HDL cholesterol, and triglycerides were further adjusted and showed TyG group 3 and VATI group 3 were associated with POAF (HR: 1.89, 95% CI 1.16–2.83 and HR: 2.61, 95% CI 1.81–4.38), but VAI group 3 was not (HR: 1.37, 95% CI 0.92–1.85).

## 4. Discussion

The present study assessed the association of CT-based mid-third lumbar vertebrae VATI, VAI, and TyG index with the development of new-onset POAF in patients after OPCABG. In our analysis we found a 1.0 SD increase in CT-based VATI, VAI, and TyG-index levels was significantly associated with an increased risk of new-onset POAF using a fully adjusted model as both a continuous or categorical variable. The sensitivity analyses conducted also confirmed that the CT-based VATI and TyG index were more independently correlated with new-onset POAF than was VAI.

Visceral adipose tissue is an important component of the body and participates in key pathophysiologic processes of regulating lipid and glucose metabolism, insulin sensitivity, and inflammation [22]. Therefore, exploration of the indexes of adiposity to better target likely indicators of CVD remains clinically significant. Previous studies found that a high level of visceral adiposity, rather than general adiposity, was significantly associated with increased CVD risk [23, 24]. A large prospective study, the Heart Outcomes Prevention Evaluation study, examined the association between MI and BMI, or abdominal obesity indexes, and found that abdominal obesity indexes were independent predictors of CVD death, MI, and total mortality [25]. Rexrode *et al*. [26] observed that elevated levels of visceral adipose tissue were independently associated with a two-fold increase in CHD risk, even after adjusting for hypertension, diabetes, and high cholesterol. In the INTERHEART study, which enrolled 27,098 participants from 52 countries, visceral adipose tissue was found to be closely correlated with the risk of CHD even after adjusting for other risk factors [27]. However, a crucial index used to represent the visceral adiposity in the INTERHEART study was waist circumference (the ratio of abdominal waist to hip circumference) which is a metric that many view as less persuasive than a CT-based visceral adipose tissue index.

The development of imaging technologies, such as CT, has yielded extraordinary progress in understanding the distribution of adipose tissue. CT can scan the whole body, or specific regions of the body, to generate cross-sectional images and calculate respective cross-sectional areas [22]. Nevertheless, there have been only a few clinical studies conducted concerning the association between CT-based VATI and CVD. In an analysis of the Framingham Heart Study Offspring cohort, Mahabadi *et al*. [23] observed that visceral abdominal adipose tissue is associated with CVD independent of traditional measures of obesity; however, the association between the CT-based VATI and new-onset POAF was not clearly established. Our data show that VATI is associated with POAF, the fully adjusted HR (95% CI) in tertile group 3 versus tertile group 1 was 2.58 (1.12–3.45), and the AUC of the VATI when added into the original model was 0.897 (*p* < 0.001). We also identified that the cutoff value of the cross-sectional VATI predictive of POAF risk was 51.34. To the best of our knowledge, this is the first report that used CT to determine the association of VATI with POAF after OPCABG. In addition, multivariate adjusted models were performed to control for important confounders and this allowed us to provide more precise estimates of the association between VATI and POAF.

In the present study, we used VAI to further investigate a potential association between visceral adipose tissue and new-onset POAF in patients after OPCABG. We found that the association between VAI and POAF was significant after adjusting for age, sex, BMI, and other variables. The fully adjusted HR (95% CI) of VAI in tertile group 3 versus tertile group 1 was 1.88 (1.21–2.95). Furthermore, VAI had a moderate predictive value for POAF with the AUC of VAI added into the original model calculated to be 0.801 (*p* = 0.021). Previous studies have found that VAI was significantly correlated with an increased risk of various CVDs. For example, Britton *et al*. [28] observed that VAI, when compared to BMI and waist circumference, was more predictive of cardiovascular risks in a study conducted on the general population. In addition, Amato *et al*. [29] reported, based on data from 1498 patients, that VAI was a more sensitive and specific predictor of cardiovascular and cerebrovascular events. In another study encompassing a large sample, the elevated level of VAI was also related to cardiovascular events [30]. However, data concerning the association between VAI and POAF after cardiac surgery are relatively limited with only a single small retrospective study that included 199 patients that had on-pump coronary artery bypass operations. Engin *et al*. [31] found that a high VAI level was associated with an increased POAF risk. Thus, our findings, when taken together with previous studies, support a close relationship between VAI and POAF in patients undergoing either off-pump or on-pump CABG. Furthermore, results suggest that visceral adipose tissue cannot be overlooked as one of the important determinants of potential illness.

Although the underlying mechanisms by which visceral adipose tissue affects atrial fibrillation have not been fully demonstrated, available studies have determined that visceral fat is closely associated with IR [15]. IR refers to the inability of endogenous insulin to be used for peripheral tissues and dysregulated glucose homeostasis in the body [15]. This may predispose patients to progression of atrial fibrillation by enlarging the left atrial size or by damaging the diastolic function of the left ventricle [32, 33, 34], activating the mitogen-activated protein kinase (MAPK) pathway to induce atrial electrical and structural remodeling [34], and destroying the function of insulin-sensitive glucose transporters [35]. The TyG index has been demonstrated to be a more convenient and valid indicator of IR than the current gold standard established using a hyperinsulinemic-euglycemic clamp because the TyG index is simpler and more cost-effective to calculate [17]. Many studies have indicated that TyG index is positively associated with cardiovascular outcomes in different patient populations [19, 20]. To date, only a limited number of studies investigating the relationship between TyG index and POAF have been performed. An analysis of 549 patients who developed ST-segment elevation MI after percutaneous coronary intervention, indicated that elevated TyG index was associated with an increased risk of new-onset atrial fibrillation [36]. Another small retrospective study, with 409 patients diagnosed with hypertrophic obstructive cardiomyopathy, found that the TyG index was an independent predictor of POAF in patients who had undergone septal myectomy [37]. No previous studies, however, have directly investigated the association between the TyG index and the occurrence of new-onset POAF after OPCABG. In the present study, patients in the highest tertile of the TyG index were more inclined to develop POAF than those in the lowest tertile. In addition, we found that a higher TyG index was an independent risk factor for POAF incidence, and had a moderate predictive value as the AUC of the TyG index, when added into the original model, was calculated as 0.878 (*p* = 0.005).

Obesity has proven to be one of the important, and modifiable, determinants of CVD and can result in undesired outcomes such as POAF after treatments such as CABG [38]. Therefore, it is clinically important to identify obese patients early who are at risk for new-onset POAF. Doing so will help to ensure that adequate precautions are taken to improve clinical outcomes. BMI is the most commonly used indicator of obesity; however, among cardiovascular risk factors, the usability of BMI is controversial and has led to the so-called “obesity paradox” [27]. Consequently, a growing number of studies indicate that excess central obesity was more useful for the prognosis of CVD and prediction of atrial fibrillation [39]. Furthermore, previous studies demonstrated that IR may play a key role in abnormal lipid deposition [15]. Nevertheless, clinical evidence concerning a potential association between visceral adiposity/IR and the complication of POAF after OPCABG has not yet been clearly established. In light of all available information, the strengths of our study include the fact that this represents the first report exploring the potential association between CT-based VATI and new-onset POAF after OPCABG. In addition, both VAI and TyG index are ideal biochemical indicators well-suited for use in routine clinical practice as these indices enable the convenient identification of patients at high risk of POAF. This study also identified cutoff values for desirable VATI, VAI, and TyG indexes and these values may be useful in predicting risk of POAF by using ROC curve analysis, as well as using the multiple-adjusted spline regression model.

There were several limitations noted in this study. First, this study is a single-center, retrospective study. Future large-scale prospective studies are needed to provide conclusive supporting evidence. Second, owing to a lack of data, we could not proceed with serial measurements of VATI, VAI, and TyG index; therefore, we were unable to investigate the correlation between index changes and POAF. Third, we did not analyze follow-up outcomes and this prevented study of the long-term effects related to VATI, VAI, and TyG index. Moreover, VAI is not a currently recognized definitive diagnostic test and it should be used with other clinical assessments to guide risk identification and treatment strategies.

## 5. Conclusions

The findings outlined in the present study suggest that elevated VATI, VAI, and TyG index values are independently associated with new-onset POAF in patients who have undergone OPCABG. Large-scale observational studies are needed to verify the present findings; however, our findings indicate that it is plausible to conclude that VATI, VAI, and TyG index may be useful measurements to use as as risk-stratification tools for patients that have undergone OPCABG.

## Data Availability

The datasets used and/or analyzed during the current study are available from the corresponding author on reasonable request.

## Supplementary Material

Supplementary material associated with this article can be found, in the online version, at https://doi.org/10.31083/j.rcm2411338.
