# Supplementary material for: Predictive Value of the CT-Based Visceral Adiposity Tissue Index and Triglyceride–Glucose Index on New-Onset Atrial Fibrillation after Off-Pump Coronary Artery Bypass Graft: Analyses from a Longitudinal Study
Source: Rev Cardiovasc Med. 2023 Nov 30;24(11):338. doi: 10.31083/j.rcm2411338 (PMC11272861; doi:10.31083/j.rcm2411338)
Supplement: Supplementary file 1 [file 2153-8174-24-11-338-s1.docx]

**Supplement Fig. 1. Distribution of new-onset atrial fibrillation following off-pump coronary artery bypass surgery in diferent subgroups.** VATI, visceral adiposity tissue index; TyG, triglyceride glucose index; VAI, visceral adiposity index.


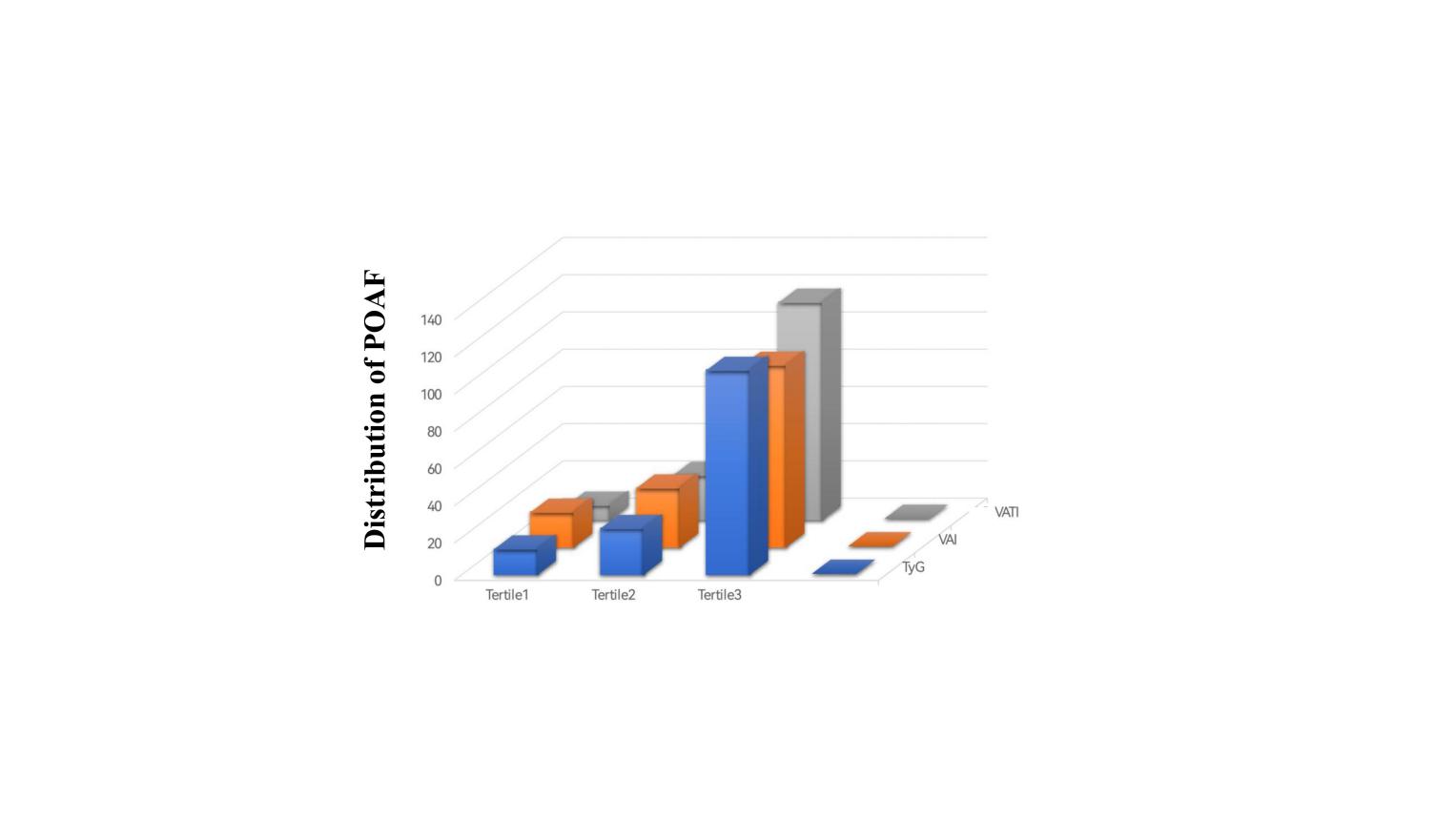


**Supplement Table 1. Clinical outcomes for of the study population.**

| **Outcomes**[%(n)] | **Visceral Adiposity Index Tertiles** | | | |  | **VATI Tertiles** | | | |  | **TyG Index Tertiles** | | | |
| --- | --- | --- | --- | --- | --- | --- | --- | --- | --- | --- | --- | --- | --- | --- |
|  | **VAI 1**  **(≤1.15 )**  **(n=180)** | **VAI 2**  **(1.15–1.66]**  **(n=184)** | **VAI 3**  **(＞1.66)**  **(n=178)** | ***P*** |  | **VATI 1**  **(≤39.72)**  **(n=180)** | **VATI 2**  **(39.72–49.84]**  **(n=182)** | **VATI 3**  **(＞49.84)**  **(n=180)** | ***P*** |  | **TyG 1**  **(≤8.45)**  **(n=181)** | **TyG 2**  **(8.45–8.80]**  **(n=185)** | **TyG 3**  **(＞8.80)**  **(n=176)** | ***P*** |
| Surgical mortality | 2.7 (5) | 1.1(2) | 3.4(6) | 0.34 |  | 3.3(6) | 0(0) | 3.9(7) | 0.03 |  | 2.8(5) | 0.5(1) | 4.0(7) | 0.09 |
| POAF | 9.9(18) | 17.0(31) | 54.5(97) | 0.01 |  | 3.9(7) | 12.6(23) | 64.4(116) | 0.01 |  | 7.2(13) | 13.0(24) | 61.9(109) | 0.01 |
| MI associated with CABG | 1.1(2) | 1.6(3) | 4.5(8) | 0.07 |  | 3.3(6) | 1.6(3) | 2.2(4) | 0.56 |  | 0.6(1) | 2.7(5) | 4.0(7) | 0.10 |
| Infection | 2.2(4) | 0.5(1) | 0.6(1) | 0.23 |  | 1.1(2) | 1.1(2) | 1.1(2) | 1 |  | 2.2(4) | 0.5(1) | 0.6(1) | 0.22 |
| Renal dysfunction | 4.9(9) | 1.6(3) | 4.5(8) | 0.19 |  | 3.3(6) | 3.3(6) | 4.4(8) | 0.81 |  | 5.0(9) | 2.7(5) | 3.4(6) | 0.50 |
| IABP support | 11.5(21) | 10.4(19) | 15.7(28) | 0.28 |  | 8.3(15) | 13.2(24) | 16.1(29) | 0.07 |  | 15.5(28) | 9.2(17) | 13.1(23) | 0.18 |
| Re-exploration for bleeding | 8.2(15) | 4.9(9) | 6.9(12) | 0.45 |  | 5.6(10) | 6.6(12) | 7.8(14) | 0.69 |  | 7.7(14) | 3.8(7) | 8.5(15) | 0.15 |
| Respiratory complication | 6.6(12) | 6.6(12) | 3.4(6) | 0.31 |  | 4.4(8) | 4.4(8) | 7.8(14) | 0.27 |  | 6.6(12) | 3.8(7) | 6.3(11) | 0.43 |
| Stroke | 3.8(7) | 5.5(10) | 4.5(8) | 0.75 |  | 2.8(5) | 6.0(11) | 5.0(9) | 0.32 |  | 4.4(8) | 5.9(11) | 3.4(6) | 0.51 |

VATI:The mid-third lumbar vertebrae visceral adiposity tissue index;TyG:Triglyceride glucose index;VAI:visceral adiposity index;POAF:Postoperative atrial fibrillation;MI:[myocardial infarction](javascript:;);CABG:coronary artery bypass grafting;IABP:intra-aortic ballon pump.

**Supplement Table 2.** **Sensitivity Analyses (HR and 95% CIs) for POAF by TyG Index Tertiles and VATI Tertiles.**

| **Model** | **TyG Index Tertiles** | **POAF** | | **VATI**  **Tertiles** | **POAF** | | **VAI**  **Tertiles** | **POAF** | |
| --- | --- | --- | --- | --- | --- | --- | --- | --- | --- |
|  |  | **HR** | **95% CI** |  | **HR** | **95% CI** |  | **HR** | **95% CI** |
| Primary model | TyG 1 | 1.0 |  | VATI 1 | 1.0 |  | VAI 1 | 1.0 |  |
|  | TyG 2 | 1.06 | 0.95-1.11 | VATI 2 | 1.26 | 0.95-1.58 | VAI 2 | 1.04 | 0.92-1.15 |
|  | TyG 3 | 1.52 | 1.12-2.75 | VATI 3 | 2.18 | 1.31-3.27 | VAI 3 | 1.26 | 0.95-1.47 |
|  |  |  |  |  |  |  |  |  |  |
| Second model | TyG 1 | 1.0 |  | VATI 1 | 1.0 |  | VAI 1 | 1.0 |  |
|  | TyG 2 | 1.04 | 0.61-2.12 | VATI 2 | 1.14 | 0.96-1.12 | VAI 2 | 1.06 | 0.74-1.12 |
|  | TyG 3 | 1.89 | 1.16-2.83 | VATI 3 | 2.61 | 1.81-4.38 | VAI 3 | 1.37 | 0.92-1.85 |

VATI:visceral adiposity tissue index;TyG:Triglyceride glucose index;POAF:Postoperative atrial fibrillation;HR:hazard ratio

Primary model = model 3 from Table 2 excluded the [left atrial diameter](javascript:;) more than 40 mm : age, sex, BMI,CRP,Diabetes,[Emergency](C:/Users/surface/AppData/Local/youdao/dict/Application/8.9.3.0/resultui/html/index.html#/javascript:;) [operation](C:/Users/surface/AppData/Local/youdao/dict/Application/8.9.3.0/resultui/html/index.html#/javascript:;),NYHA III–IV

Second model: Primary model + hypertension, total cholesterol, HDL cholesterol, and triglycerides
